# A novel androgen receptor gene splice site mutation induces aberrant mRNA splicing and internal in-frame deletion in androgen insensitivity syndrome

**DOI:** 10.1186/s12920-026-02374-x

**Published:** 2026-04-22

**Authors:** Baoqiong Liao, Mei Shuai, Lin Xiao, Gungao Huang, Ying He, Ya-Long Wang, Hao Wang, Shuwen He

**Affiliations:** 1 Ganzhou Maternal and Child Health Hospital, Ganzhou, Jiangxi China; 2 Loke Centre for Trophoblast Research, Department of Physiology, Development, and Neuroscience, University of Cambridge, CB2 3EG Cambridge, UK; 3 Department of Chemistry and Molecular Biology, Gothenburg University, Gothenburg, Sweden; 4 Department of Infectious Diseases, Institute for Biomedicine, Sahlgrenska Academy, University of Gothenburg, Gothenburg, Sweden; 5 Center for Reproductive Medicine, Maternity and Child Health Care Hospital in Xiangtan, Xiangtan, China

**Keywords:** Androgen receptor, Androgen insensitivity syndrome, Mutation, Whole-exome sequencing, MRNA splicing

## Abstract

Androgen insensitivity syndrome (AIS) is a disorder caused by pathogenic mutations in the androgen receptor (AR) gene, leading to androgen resistance and impaired sex differentiation in 46,XY individuals. Despite increasing recognition of AR mutations, the pathogenic mechanisms and genotype–phenotype correlations of many variants remain incompletely characterized. In this study, we identified a novel splice site mutation in the AR gene, c.2450-1G > A, in a 46,XY patient presenting with a female phenotype, including primary amenorrhea and classical features of AIS, confirmed by chromosomal analysis and ultrasonography. Functional validation using minigene splicing assays demonstrated that this variant disrupts normal splicing, leading to an aberrant transcript lacking 6 bp at the 5′ end of exon 7. This results in an aberrant in-frame transcript, c.2450_2455del, encoding a mutant protein with a three-amino acid deletion and a methionine insertion (p.Ile817_Val819delinsMet), while preserving the reading frame. In silico structural modeling suggested that this alteration may disturb the ligand-binding domain (LBD) conformation and receptor functionality. These findings highlight the pathogenic significance of splice-site variants in AR and provide mechanistic insight into AIS diagnosis and management.

**Supplementary Information:**

The online version contains supplementary material available at 10.1186/s12920-026-02374-x.

## Introduction

The androgen insensitivity syndrome (AIS) (MIM: 300,068) is one of the most common congenital disorders linked to the X chromosome and is inherited in a recessive manner [1]. It occurs in individuals with a 46, XY karyotype and results in varying degrees of disruption in sexual differentiation due to a partial or complete failure of tissues to respond to androgens [2]. Phenotypically, individuals exhibit typical female external characteristics, but lack female internal reproductive organs, thereby precluding the ability to conceive or sustain a pregnancy. Clinically, AIS is categorized based on the degree of external genital feminization into complete (CAIS), partial (PAIS), and mild (MAIS) androgen insensitivity syndrome [3, 4]. AIS may result in severe psychological distress in patients, particularly in those reared as females with CAIS or PAIS [5]. Inaccurate diagnosis or inappropriate treatment may elevate the risk of testicular tumor development and result in pubertal masculinization in PAIS, potentially resulting in the gender dysphoria [6]. Therefore, an in-depth investigation into the pathogenesis of AIS is crucial for improving clinical diagnosis and treatment of the disease.

Significant advances have been made in elucidating the molecular mechanisms underlying AIS during sexual development. A well-established cause of androgen resistance is pathogenic mutation in the androgen receptor (AR) gene (MIM: 313,700) [7]. Approximately 80–100% of individuals with CAIS harbor mutations in the AR gene [8], while the mutation frequency in PAIS cases can be as low as 16% [9]. The AR gene is located in Xq11-12 comprises eight exons, and encodes a 920-amino acid protein. The AR protein contains four key functional domains: the N-terminal domain (NTD), DNA-binding domain (DBD), ligand-binding domain (LBD), and a hinge region linking the DBD and LBD [10, 11].

As a steroid hormone-activated transcription factor, AR is essential for male sexual differentiation during embryogenesis and secondary sexual characteristic development during puberty [12]. AR mediates the essential actions of androgens, including testosterone (T) and dihydrotestosterone (DHT) [13]. Upon androgen binding, the AR dissociates from heat shock proteins (Hsps), translocates to the nucleus, and binds to androgen response elements in target gene promoters to regulate transcription [14]. With advances in sequencing technologies, numerous pathogenic variants of the AR gene have been documented in the Human Gene Mutation Database (HGMD), with point mutations being the most prevalent. In CAIS and PAIS, missense mutations frequently occur in exons encoding the DBD and LBD, impairing either androgen binding or DNA interaction, thereby disrupting downstream androgen signaling [15, 16]. However, genotype–phenotype correlation remains poorly defined, as identical mutations may result in variable clinical presentations, according to the AR mutation database (https://androgendb.mcgill.ca/).

The timing of gonadectomy in AIS patients is critical to balance the initiation of spontaneous puberty and the prevention of virilization. Functional analysis of AR variants is essential for informing early clinical strategies, enabling personalized treatment plans that optimize patient outcomes. In this study, we identified a novel splice-site mutation in the AR gene, c.2450-1G > A, in a patient exhibiting classical AIS phenotypes, confirmed through comprehensive clinical and genetic evaluation. Our findings highlight the significant pathogenic impact of non-coding splice-site mutations within the AR gene, deepening our understanding of its molecular mechanism and its role in the pathogenesis of AIS. This mechanistic understanding will contribute to improve diagnostic accuracy and tailor therapeutic interventions for affected individuals.

## Materials and methods

### Patient samples and DNA extraction

This study received approval from the Ethics Committee of the Ganzhou Maternal and Child Health Hospital, Jiangxi, China. After obtaining informed consent, peripheral venous blood samples were collected from the patient as previously described [17]. Genomic DNA extraction was carried out using the QIAamp DNA Blood Midi Kit (QIAGEN) according to standard procedures. Purity and concentration of DNA were assessed spectrophotometrically and confirmed by agarose gel electrophoresis. Only high-quality DNA was used for subsequent library preparation, utilizing the FreySeq® Whole Exome Library Preparation and Hybridization Capture Kit (V2.0 plus + mt) from Fuzhou Frey Medical Laboratory Co., Ltd., which targets specific genomic regions for enrichment.

### Whole exome sequencing

Sequencing libraries passing QC were subjected to paired-end sequencing (150 bp reads) on an Illumina NovaSeq 6000 platform, achieving ≥ 96% of targeted regions covered at ≥ 20 ×. Raw reads were mapped to the GRCh37/hg19 human genome reference. Variant calling included SNVs and small InDels with coverage ≥ 10 ×. CNV analysis was performed using CNVexon™ software (Fulgent Technologies Inc.) focusing on genes with clinical relevance. Variant interpretation was supported by population and clinical databases (dbSNP, 1000 Genomes, ExAC, OMIM, HGMD, ClinVar) and predictive algorithms (SIFT, PolyPhen-2, Mutation Taster, SpliceAI). SpliceAI and Pangolin predicted an acceptor loss for the variant chrX-67722826 G > A with scores of 0.99 and 0.82 at position 1 bp, and an acceptor gain with scores of 0.89 and 0.78 at position 7 bp. Splice impact was further evaluated via RDDCSC software (https://rddc.tsinghua-gd.org/tool/rna-splicer). A novel AR gene splice-site variant (c.2450-1G > A) was identified and validated by Sanger sequencing.

### Plasmid constructs for splicing assay

To investigate the splicing effects of the AR c.2450-1G > A variant, genomic fragments were amplified from wild-type and mutant templates. PCR amplification was performed under the following conditions: initial denaturation at 95 °C for 3 min; 35 cycles of 95 °C for 30 s, 57 °C for 30 s, and 72 °C for 1 min; followed by a final extension at 72 °C for 5 min.

For the cMINI-N construct, a fragment containing exon 6 (131 bp), intron 6 (863 bp), exon 7 (158 bp), and part of intron 7 (434 bp) was cloned into the pcMINI-N vector containing an MCS–intron B–exon B cassette [18]. For the pcDNA3.1 construct, a fragment including exon 6 (131 bp), intron 6 (863 bp), exon 7 (158 bp), intron 7 (701 bp), and exon 8 (156 bp) was inserted into the pcDNA3.1 vector (Baiyi Biotech Co., China. Fragments were ligated at 22 °C for 90 min using T4 DNA ligase, followed by transformation into DH5α competent cells. Positive clones were confirmed by sequencing.

### Cell culture and transfection

HeLa and HEK293T cells (obtained from Wuhan Baiyi Biotechnology Co., Ltd., China) were cultured in Dulbecco’s Modified Eagle Medium (DMEM) supplemented with 10% fetal bovine serum and 1% penicillin–streptomycin at 37 °C with 5% CO₂. Cells were seeded at approximately 1–2 × 10^5^ cells per well in a 6-well plate and cultured for 1–3 days until reaching 70–80% confluency. They were then transiently transfected with 2 μg of each minigene construct using 2ul Lipofectamine 3000 (Thermo Fisher Scientific, USA) according to the manufacturer’s protocol. After 48 h, cells were harvested for RNA extraction.

### RNA extraction and RT-PCR

Total RNA was isolated using the RNeasy Mini Kit (Qiagen) following the manufacturer’s instructions. RNA quality and concentration were assessed by NanoDrop spectrophotometry. Reverse transcription was performed using the PrimeScript RT reagent kit (Takara Bio) with random hexamer primers.

Minigene transcripts were amplified by PCR using vector-specific primers targeting the flanking regions of the inserted fragment (pcMINI-N-F/R and pcDNA3.1-F/R). PCR products were resolved on 2% agarose gels, purified, and sequenced to determine splicing outcomes.

### Protein structure prediction

The canonical AR protein sequence (920 amino acids) was retrieved from UniProt website (https://www.uniprot.org/). Using AlphaFold web server, 3D structure of wild-type and mutant AR proteins were predicted, selecting the top-ranked models by pLDDT confidence scores. Visualization and comparative analysis were performed in PyMOL (https://pymol.org/).

## Results

### Identification of the novel AR splice site variant

**Fig. 1:**
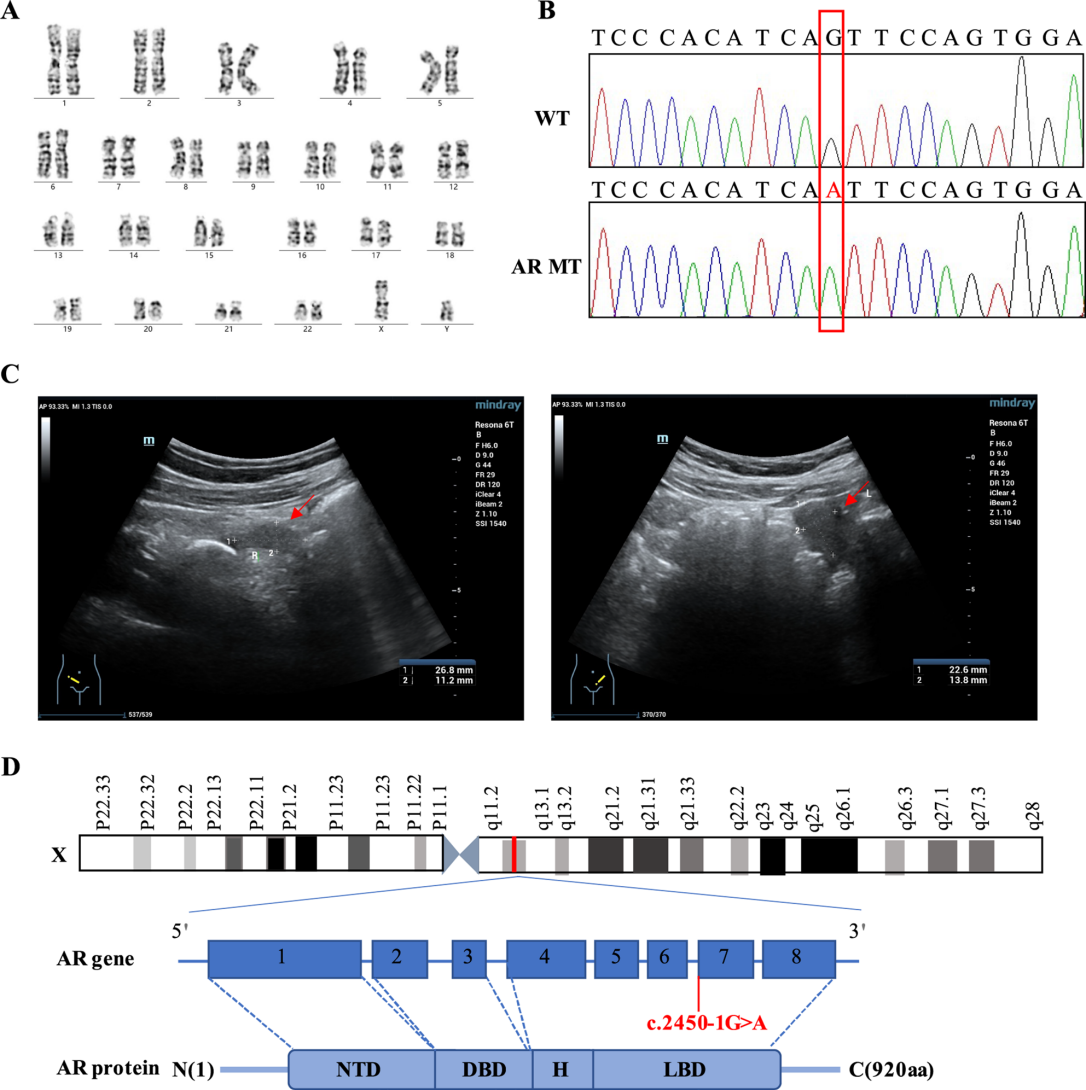
**A** Chromosome analysis results of patient with 46,XY karyotype. **B** Sanger sequencing chromatograms confirming the AR variant and providing definitive evidence of the c.2450-1G > A nucleotide alteration. **C** Pelvic ultrasonography revealing bilateral inguinal masses consistent with undescended testes. **D** Schematic representation of the AR gene illustrating the genomic location of the c.2450-1G > A variant within intron 6 and the ligand-binding domain (LBD)

At 14 years old, the patient was admitted to the hospital because of a primary amenorrhea, and phenotypically had a female appearance. Cytogenetic analysis identified a 46,XY karyotype. The patient's WES uncovered a novel mutation c.2450-1G > A in the AR gene (Fig. 1A-B), and it has not been reported in any public population databases. Hormonal evaluation revealed elevated gonadotropin levels: Follicle-stimulating hormone (FSH) at 19.5 IU/L (reference range: 3–10 IU/L), luteinizing hormone (LH) at 33.1 IU/L (reference range: 2–15 IU/L), estradiol at 101 pmol/L (reference range: 70–500 pmol/L), prolactin at 381 mIU/L (reference range: < 500 mIU/L in non-pregnant women), and an elevated testosterone level of 12.2 nmol/L for women but within the normal male range (reference range: 9–30 nmol/L in men and 0.5–2.4 nmol/L in women). Pelvic ultrasonography revealed bilateral inguinal masses consistent with testicular tissue. The vaginal canal ended in a blind pouch, and neither the uterus nor ovaries were visualized. Structures within both inguinal canals were identified: the right side measured 22.8 × 11.2 mm and the left side 22.6 × 13.8 mm, consistent with undescended testes (Fig. 1C).

The AR c.2450-1G > A mutation is located in intron 6 at the canonical −1 G splice acceptor site, resulting in an delins (p.Ile817_Val819delinsMet) in exon 7 that disrupts the ligand-binding domain (LBD) and impairs receptor function (Fig. 1D). In accordance with the American College of Medical Genetics and Genomics (ACMG) guidelines [19] for variant interpretation, the c.2450-1G > A variant meets the criteria for classification as likely pathogenic.Functional studies using a minigene assay demonstrated that c.2450-1G > A disrupts normal splicing, providing strong experimental support for a deleterious effect (PS3_Strong). The variant is absent from population databases, fulfilling the PM2 criterion, and multiple in silico splice-site prediction tools, including RNA-Dependent DNA Cleavage and Splicing Calculation (RDDCSC), SpliceAI, and MaxEntScan, consistently predict exon skipping or deletion due to this mutation, indicating a deleterious impact on RNA splicing (PP3, Supplementary Fig. 1). We further reviewed the reported splice-site mutations in intron 6 of AR gene, including c.2450-118A > G, c.2450-44G > A, c.2450-42G > A, c.2450-6C > G, and c.2450-1G > C (Table 1). All affected individuals exhibited AIS, though with varying phenotypic severity.

**Table 1:** Splicing mutations described in the AR gene (from the Human Gene Mutation Database)

Mutation	HGMD access ID	Intron	Mutation type	PMID	Pathogenicity
2450-118A > G	CS1612803	IVS6 as A-G −118	Splice donor variant	[20]	Pathogenic
2450-44G > A	CS102224	IVS6 as G-A −44	Splice donor variant	[9]	Pathogenic
2450-42G > A	CS181835	IVS6 as G-A −42	Splice donor variant	[21]	Likely pathogenic
2450-6C > G	CS1810288	IVS6 as C-G −6	Splice donor variant	[22]	Pathogenic
2450-1G > C	CS2255059	IVS6 as G-C −1	Splice donor variant	[23]	Pathogenic
2450-1G > A	ND	IVS6 as G-A-1	Splice donor variant	ND	Likely Pathogenic

### Splicing analysis

**Fig. 2:**
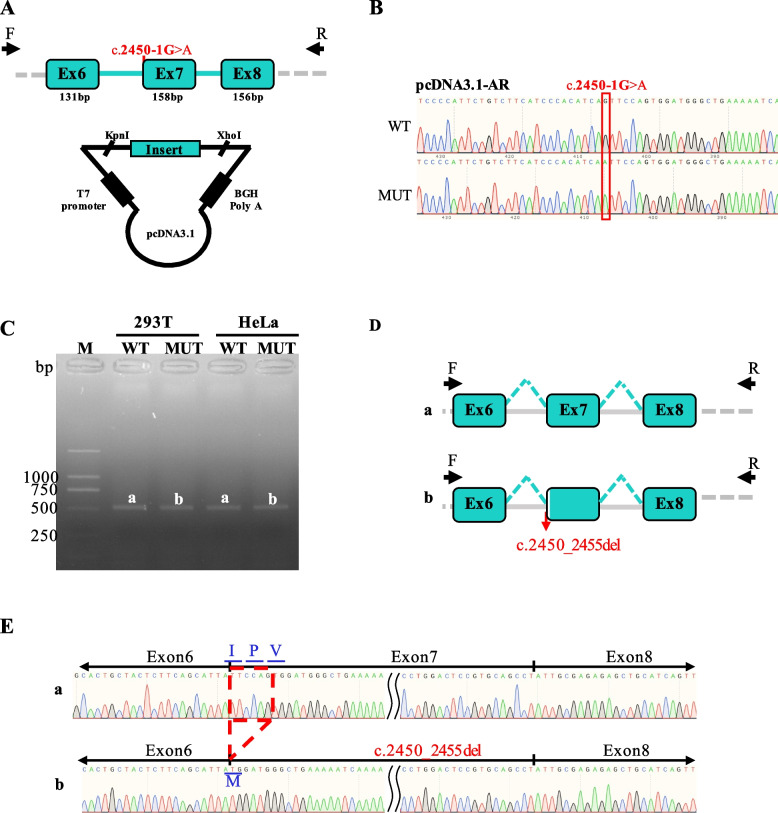
Characterization of the effect of c.2450-1G > A mutation on AR splicing. **A** Construction of the pcDNA3.1-AR-WT/MUT vector, which contain exon 6–8 and flanking intronic sequences of WT or mutant type (c.2450-1G > A) of the AR gene. **B** Sequencing confirmation of the inserted wild-type and mutant AR fragments in the recombinant vectors. **C** Minigene assay performed in HEK 293 T and Hela cells transfected with pcDNA3.1-AR-WT/MUT vector. RT-PCR was performed to amplify the AR transcript encompassing exons 6–8. Agarose gel electrophoresis shows distinct band patterns for wild-type and mutant constructs. **D** Schematic representation of the AR genomic region between exon 6 and exon 8. Arrows indicate the positions of primers used for RT-PCR (Ex6F and Ex8R). a. Wild-type exon arrangement. b. Disruption of the canonical splice acceptor site at exon 7 caused by the c.2450-1G > A variant. **E** Sanger sequencing of RT-PCR products from 293 T cells transfected with WT (a) and MUT (b) pcDNA3.1-AR constructs

To investigate the functional consequence of the AR c.2450-1G > A variant, in vitro minigene splicing assays were conducted using both pcDNA3.1 and pcMINI-N vector systems (Fig. 2A-2B, Supplementary Fig. 2). RT-PCR and subsequent sequencing revealed a consistent splicing abnormality across both constructs, characterized by a deletion of six nucleotides at the 5′ end of exon 7 (Fig. 2C-2E). This aberrant splicing event was absent in wild-type controls, indicating that the c.2450-1G > A variant directly disrupts normal exon recognition and splicing fidelity. At the transcript level, the altered splicing is denoted as c.2450_2455del, and at the protein level as p.Ile817_Val819delinsMet. This results in an in-frame deletion of three amino acids accompanied by the insertion of a methionine residue. Importantly, the mutation does not disrupt the downstream reading frame, and the predicted translation product is a near full-length androgen receptor protein. However, the alteration occurs within the ligand-binding domain (LBD), raising the possibility of compromised receptor conformation or function.

### Effect of mutation on protein structure

**Fig. 3:**
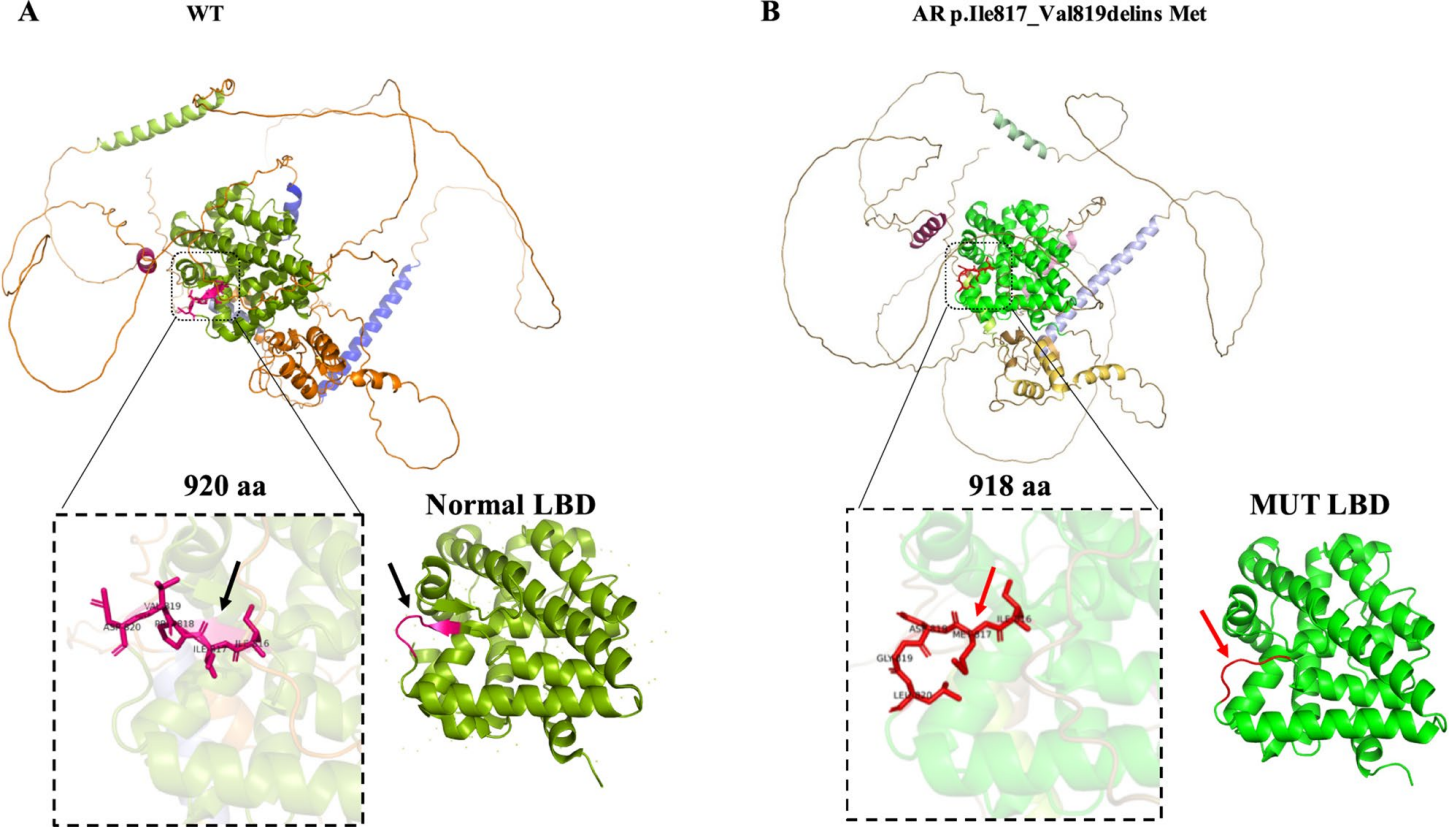
Potential impact of the c.2450-1G > A mutations on AR protein structure. **A** The wide-type structure of the AR protein was downloaded from the PDB database. The mutant protein structure shown in **B** contained a truncated AR protein that consist 918 of the 920 amino acids of the mature protein. Arrows indicate β-sheets, and green coloring highlights the ligand-binding domain (LBD)

To assess the structural consequences of the AR splice-site variant NM_000044.6: c.2450-1G > A, we utilized the AlphaFold web server to predict changes in the AR protein structure. This variant affects the canonical splice acceptor site at the intron 6-exon 7 junction and has been experimentally shown to cause aberrant splicing, resulting in the in-frame protein alteration p.Ile817_Val819delinsMet. Structural modeling indicates that the β-sheet present in the wild-type protein is completely absent in the variant protein. The loss of this β-sheet likely destabilizes the local secondary structure within the LBD,which spans approximately residues 670 to 920. In Figs. 3A and 3B, the LBD is highlighted in green for the WT and MT structures, respectively. The mutant structure was predicted to differ substantially from the wild-type. Such a disruption may impair the receptor’s ability to undergo essential ligand-induced conformational changes, thereby compromising its normal function (Fig. 3A-B). These predicted structural alterations provide the evidence supporting the possible pathogenicity of the c.2450-1G > A variant and emphasize the importance of evaluating non-frameshift splice-site mutations within critical functional domains.

## Discussion

Sex differentiation in humans is a complex, multistage process that begins in embryogenesis and continues through puberty. This process is regulated by multiple factors, with AIS being the most common cause of disorders of sex development in individuals with a 46,XY karyotype [24]. AIS is most frequently caused by mutations that lead to loss-of-function in the AR gene [25], including substitutions, translocations, deletions, or insertions. While mutations in exon 1 account for approximately 25% of AR defects, most pathogenic variants cluster within the LBD region, which is essential for receptor function [26]. The AR mutations identified in a subset of CAIS cohorts were single-nucleotide missense variants localized within the LBD, suggesting the functional importance of this region [27].

The proband in this study exhibited a completely female external phenotype. Upon further investigation of the proband’s family, no clinically affected individuals were identified. We identified and functionally characterized a novel splice-site variant, c.2450-1G > A, in the AR gene, which has not been previously reported. This variant aligns with the clinical features observed in the patient. Our findings reveal that this intronic mutation disrupts the canonical splice acceptor site upstream of exon 7, resulting in an aberrant transcript lacking six nucleotides at the 5′ end of the exon. The altered transcript produces an in-frame deletion at the cDNA level (c.2450_2455del) and a corresponding protein alteration (p.Ile817_Val819delinsMet), which retains the original reading frame but deletes two conserved amino acids within the LBD of the AR protein and changed valine 819 to methionine.

Functionally, LBD is essential for high-affinity binding of dihydrotestosterone (DHT) and proper nuclear translocation of the AR, which are critical steps for transcriptional activation of androgen-responsive genes [28, 29]. Thus, even subtle structural alterations within this region can significantly impair receptor function [30]. Importantly, pharmacological agents that inhibit AR activity act by binding to its LBD, thereby slowing disease progression [31, 32]. Structural modeling predicted that the resulting amino acid deletion and methionine insertion disrupt local secondary structures, likely altering ligand interaction dynamics and receptor conformation. This is consistent with previous observations that LBD variants compromise AR activity and lead to androgen insensitivity [33–35]. However, we recognize that these predictions are speculative and highlight the need for future experimental structural validation to confirm the proposed effects.

Previous studies have demonstrated that not all AR mutations causing AIS result in complete loss of function. Earlier studies reported that up to 33.3% of individuals with CAIS and 58.7% of those with PAIS did not exhibit identifiable AR mutations [36]. However, more recent exome and genome sequencing analyses have increased the detection rate in CAIS to approximately 85–95% [37]. In some cases, residual receptor activity remains, particularly under high androgen concentrations, which may contribute to variable phenotypic expression, including partial virilization during puberty. Mutations such as Asp864Asn and Leu907Phe exhibited considerable androgen-binding and transactivation capabilities despite being associated with CAIS phenotypes [38]. Although our study did not directly assess androgen response in vitro, mutations affecting the ligand-binding domain of AR have been associated with a broad spectrum of AIS phenotypes, ranging from partial to complete androgen insensitivity. Importantly, a missense mutation at a neighboring residue (p.Pro818Leu) has been reported in individuals with complete AIS [39], underscoring the functional importance of this region and suggesting that alterations at or near this site can result in severe receptor dysfunction. Therefore, while residual AR activity cannot be excluded without direct functional assays, the location of the c.2450-1G > A–induced protein alteration within the ligand-binding domain is consistent with the complete androgen insensitivity phenotype observed in our patient.

Unresolved challenges in the clinical management of AIS include decisions regarding reconstructive surgery, genetic counseling, gender assignment, timing of gonadectomy, long-term reproductive, and physiological outcomes [40, 41]. While advances in genomic technologies have enhanced our understanding of AIS pathogenesis, diagnosis remains complex, and a definitive molecular diagnosis is not always possible. The correlation between AR genotype and clinical phenotype remains poorly defined. Therefore, expanding the spectrum of identified AR mutations is crucial for guiding clinical decision-making. Our research further emphasizes the clinical relevance of non-coding mutations, which may be overlooked by exome sequencing alone. Comprehensive genetic analysis, including intronic regions flanking exons, is essential in suspected AIS cases, especially when coding-region variants are not detected. Functional studies, such as direct in vitro evaluation of androgen binding affinity and AR transactivation capacity, will help to clarify its effect on receptor-ligand interaction and downstream signaling activity.

In conclusion, we report a novel AR splice-site mutation, c.2450-1G > A, that results in an internally truncated, structurally altered AR protein likely contributing to androgen insensitivity. Our findings support the use of minigene splicing assays and structural analysis in the evaluation of non-coding variants and provide mechanistic insight into how subtle splice disruptions can contribute to AIS. These results have important implications for molecular diagnosis, genotype–phenotype prediction, and personalized clinical management in patients with disorders of sex development.

## Supplementary Information

Supplementary Material 1. Supplementary Figure 1 RDDCSC analysis predicts that the mutation leads to usage of a new splice acceptor site and loss of 6 nt in exon 7 (splice pattern 1) or skipping of exon 7 (splice pattern 2), suggesting that the variant affects pre-mRNA splicing.

Supplementary Material 2. Supplementary Figure 2 (A) Construction of the pcMINI-N-AR-WT/MUT vector, which contain exon 6 and 7 and Exon B (ASL exon 4). Sequencing confirmation of the inserted wild-type and mutant AR fragments in the recombinant vectors. (B) Minigene assay performed in HEK 293T and Hela cells transfected with pcMINI-N-AR-WT/MUT vector. RT-PCR was performed to amplify the AR transcript encompassing exon 6 and 7 and the vector derived exon B. (C) Sanger sequencing of RT-PCR products from 293T cells transfected with WT (a) and MUT (b) pcMINI-N-AR constructs.

## Data Availability

The variant data generated and analysed during the current study has been deposited in the ClinVar database and is publicly available under the following accession: VCV000419726.2 (Variation ID: 419,726). Additional datasets used in this study are available from the corresponding author upon reasonable request (shuwen.he@gu.se).
