# Supplementary material for: A novel androgen receptor gene splice site mutation induces aberrant mRNA splicing and internal in-frame deletion in androgen insensitivity syndrome
Source: BMC Med Genomics. 2026 Apr 22;19:94. doi: 10.1186/s12920-026-02374-x (PMC13238024; doi:10.1186/s12920-026-02374-x)

Human AR (NM_000044.6, c.2450-1G>A)

Splice Pattern 1:

Deleting 6bp, new acceptor

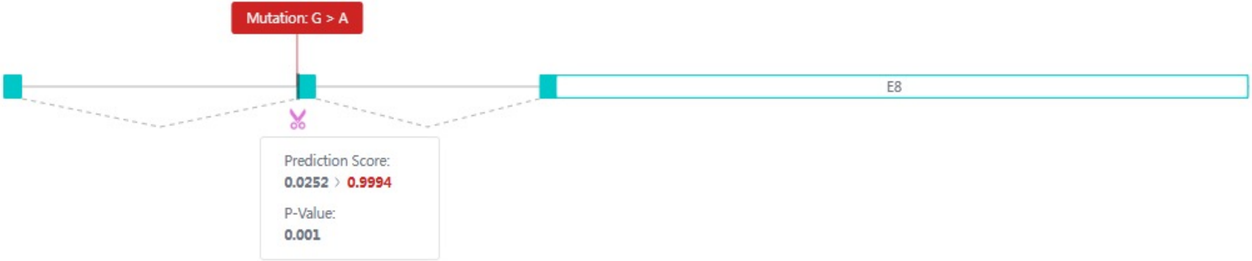

Splice Pattern 2:

Deleting 158bp, exon skipping, frameshift mutation, premature termination

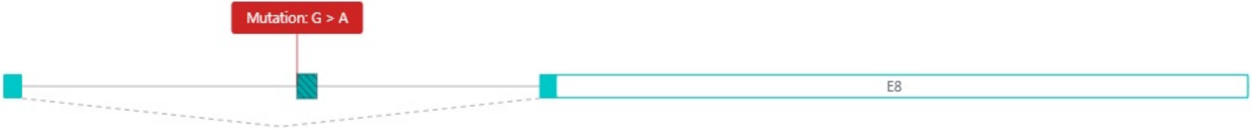

Supplement: Supplementary file 1 — Supplementary Material 1. Supplementary Figure 1 RDDCSC analysis predicts that the mutation leads to usage of a new splice acceptor site and loss of 6 nt in exon 7 (splice pattern 1) or skipping of exon 7 (splice pattern 2), suggesting that the variant affects pre-mRNA splicing. [file 12920_2026_2374_MOESM1_ESM.pdf]
