# Supplementary material for: A novel androgen receptor gene splice site mutation induces aberrant mRNA splicing and internal in-frame deletion in androgen insensitivity syndrome
Source: BMC Med Genomics. 2026 Apr 22;19:94. doi: 10.1186/s12920-026-02374-x (PMC13238024; doi:10.1186/s12920-026-02374-x)
Supplement: Supplementary file 2 — Supplementary Material 2. Supplementary Figure 2 (A) Construction of the pcMINI-N-AR-WT/MUT vector, which contain exon 6 and 7 and Exon B (ASL exon 4). Sequencing confirmation of the inserted wild-type and mutant AR fragments in the recombinant vectors. (B) Minigene assay performed in HEK 293T and Hela cells transfected with pcMINI-N-AR-WT/MUT vector. RT-PCR was performed to amplify the AR transcript encompassing exon 6 and 7 and the vector derived exon B. (C) Sanger sequencing of RT-PCR products from 293T cells transfected with WT (a) and MUT (b) pcMINI-N-AR constructs. [file 12920_2026_2374_MOESM2_ESM.docx]

**Figure 2C: pcDNA3.1-AR**

**b**

**b**

**a**


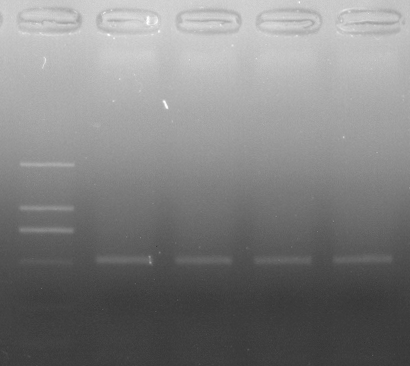


250

500

750

1000

**WT**

**MUT**

**WT**

**MUT**

**293T**

**HeLa**

bp

**b**

**b**

**a**

**M**

**a**

**a**

**Supplementary Figure 2B**: **pcMINI-N-AR**

**b**

**b**

**a**


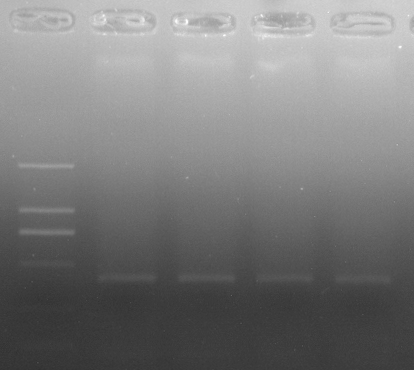


250

500

750

1000

**HeLa**

**MUT**

**MUT**

**WT**

**WT**

**293T**

**M**

**b**

**a**

**b**

**a**

bp
